# Very high power short duration as re-ablation strategy for recurrent atrial fibrillation

**DOI:** 10.1186/s12872-025-05448-3

**Published:** 2025-12-30

**Authors:** Jana Ackmann, Jonas Wörmann, Jakob Lüker, Jan-Hendrik Schipper, Jan-Hendrik van den Bruck, Cornelia Scheurlen, Sebastian Dittrich, Friederike Pavel, Theodoros Maximidou, Daniel Steven, Arian Sultan

**Affiliations:** 1 Department of Electrophysiology, Heart Center, University of Cologne, Kerpener Str. 62, Cologne, 50937 Germany; 2 Asklepios Hospital St. Georg, Heart Center, Lohmühlenstrasse 5, Hamburg, 20099 Germany

**Keywords:** PVI, Catheter ablation, Atrial fibrillation, Radiofrequency ablation, Re-ablation, High-Power Short-Duration (HPSD)

## Abstract

**Background:**

After initially successful pulmonary vein isolation (PVI), some patients experience recurrence of AF requiring repeat catheter ablation. Radiofrequency ablation (RFA) is then mostly performed with low power long duration settings (LPLD), while very high power short duration (vHPSD) may be a valuable alternative. This study aimed to compare procedural parameters, outcomes and complication rates of both approaches.

**Methods:**

All consecutive repeat procedures for recurrent AF between 12/2019 and 03/2023 were retrospectively analyzed at our center. Only patients with at least one prior ablation were included. RFA was performed using LPLD (≤ 40 W) or vHPSD (≥ 70 W, 7 s). Ablation strategies were at the operators’ discretion.

**Results:**

A total of 340 procedures were included (LPLD: 292; vHPSD: 48). Re-PVI was necessary in the majority of cases (LPLD: 67.8%; vHPSD: 70.8%; *p* = 0.7). Additional ablation lines were performed in 59.9% (LPLD) and 75.0% (vHPSD; *p* = 0.1). In the vHPSD group more patients underwent substrate modification (CFAE ablation/scar encirclement; LPLD: 27.7%; vHPSD: 45.8%; *p* = 0.017). Despite this, RF delivery time was shorter with vHPSD (LPLD: 2024 ± 1014 s; vHPSD: 1591 ± 936 s; *p* = 0.006), while total procedure duration (LPLD: 142.8 ± 45.8 min; vHPSD: 139.2 ± 48.2 min; *p* = 0.7) and complication rates (LPLD: 5.1%; vHPSD: 8.3%; *p* = 0.3) were similar. After a median follow-up of 361 days, arrhythmia-free survival was comparable between groups (LPLD: 65.7%; vHPSD: 68.8%; *p* = 0.8).

**Conclusion:**

vHPSD is an efficient alternative to conventional RFA in repeat procedures for recurrent AF, even in complex cases requiring extended lesion sets.

## Introduction

Pulmonary vein isolation (PVI) is the cornerstone for treatment of atrial fibrillation [1]. However, recurrence of atrial fibrillation (AF) might occur and patients require repeat catheter ablation (CA) also potentially targeting areas outside the pulmonary veins. For repeat CA standard radiofrequency (RF) in the setting of a 3D mapping system is the most prevalent used modality.

Commonly, conventional RF CA uses a low power long duration (LPLD) setting (< 50 watts (W) over a longer time period) or is index guided (ablation index, AI [2–4]/lesion index, LSI [5, 6]). This approach was challenged using RF in a very high power short duration (vHPSD) mode. Multiple studies proved safety and efficacy of vHPSD settings for PVI [7–9]. The term HPSD in general is used widely, however HPSD is mostly defined by energy settings of > 50 W. Short vHPSD impulses of 7 s (5 s at the posterior wall) with a power setting of 70 W create more shallow but broader lesions [10] due to resistive heating compared to LPLD settings creating deeper but narrower lesions (conductive heating). Therefore, vHPSD has the potential to achieve thorough and continuous ablation lines with potentially improved outcome and reduced ablation time [8]. The specific physics of different energy settings and lesion formation has been described previously [10].

Although most studies have demonstrated the safety of vHPSD settings for PVI, concerns remain regarding steam pops associated with embolic events, pericardial tamponades, and thermal esophageal injury. Data on the use of vHPSD settings in atrial ablation beyond PVI are sparse. Therefore, this study was initiated to compare feasibility, safety and outcome of vHPSD with LPLD settings in repeat ablation for AF.

## Methods

### Study design

We conducted a retrospective study of re-ablation procedures for AF between December 2019 and March 2023 at the University Hospital Cologne. Patients who underwent CA with either LPLD or vHPSD settings as described below were included. A follow-up was conducted 3 and 12 months after the procedure and included at least one of the following: Holter-ECG, 12-lead ECG, app-based telemonitoring or device interrogation if applicable. An electronic database (RedCap, Research Electronic Data Capture [11]) was used for data acquisition. Documentation of AF, AT or atrial flutter over 30 s was defined as recurrence of atrial arrhythmia. All patients provided written informed consent for the procedure and data analysis.

### Ablation procedure

In all procedures deep analgo-sedation was applied using fentanyl, midazolam and propofol. Direct oral anticoagulants (DOACs) with one daily dose were discontinued the day prior to ablation and DOACs with two daily doses were discontinued the evening prior to ablation. Vitamin K antagonists were continued with an international normalized ratio (INR) of 2—3 considered suitable for ablation. Patients who had no continuous intake of anticoagulants and a CHA_2_DS_2_-VASc-Score ≥ 2 or showed atrial arrhythmia in the beginning of the procedure received preprocedural transesophageal echocardiography (TEE) to exclude intracardiac thrombi [12]. Venous access was gained via the right femoral vein. A catheter (Dynamic XT™, Boston Scientific, Marlborough, MA, USA) was placed into the coronary sinus (CS) as reference. Transseptal puncture (TSP) was then performed under fluoroscopic guidance (TSX™ fixed curve transseptal sheath, TSX™ transseptal needle, Boston Scientific). Following TSP weight-adjusted boli of unfractionated heparin (UFH) were administered to achieve an activated clotting time (ACT) over 300 s. ACT measurement and ACT-adjusted UFH administration were repeated every 30 min.

In all procedures a 3D mapping system (Ensite NavX™/Ensite Precision™/Ensite X™; Abbott, St. Paul, MN, USA or CARTO®; Biosense Webster, Irvine, CA, USA) was employed. Voltage mapping was performed in the rhythm the patient presented with during the procedure. The LA scar threshold was set at 0.2 mV. Durable PVI was evaluated by high-density (HD) mapping and proof of entrance and exit block (if in SR). Exit block was confirmed by pacing at the antral aspect of each pulmonary vein, using fluoroscopy and electrogram quality to ensure adequate tissue contact. Ablation catheters were the ThermoCool® SmartTouch® Surround Flow (ST-SF; Biosense Webster) and TactiCath™ (Abbott) with contact force measurement or FlexAbility™ (Abbott) ablation catheter without contact force measurement. A maximum RFA energy of 40 W managed by ablation index (AI [2–4], Biosense Webster) or lesion index (LSI [5, 6], Abbott) was considered LPLD. For vHPSD ablation settings were 70 W for 7 s with exception of the posterior wall where settings were 70 W for 5 s. In procedures performed without contact force sensing, tissue contact was assessed using catheter stability, electrogram attenuation, impedance drop, and fluoroscopic catheter position. If reconnection of PVs was proven re-PVI was performed. Lesions were applied with visually contiguous antral spacing, aiming for an interlesion distance of approximately ≤ 6 mm, as suggested by CLOSE-based approaches [13]. Further ablation strategy beyond re-PVI was at operator´s discretion. If typical atrial flutter was observed ablation of the cavotricuspid isthmus (CTI) was performed with LPLD settings in both groups.

For femoral access closure, a figure-of-eight suture was performed, followed by the application of a compression bandage for 6 h. All patients underwent focused transthoracic echocardiography (TTE) to exclude pericardial effusion immediately after the procedure, 2 h post-procedure, and the following morning. Procedure time was defined as the interval from skin puncture to sheath removal ("skin-to-skin" time). Continuous ECG monitoring was conducted for 48 h, and patients were typically discharged 48 h after the procedure.

### Statistical analysis

Statistical analysis was performed with Microsoft Excel for Mac (version 16.86) and Prism 10 for MacOS (version 10.2.3). Continuous variables are shown as mean (± SD). A Gaussian distribution was tested with the Shapiro–Wilk test and analysis was performed either with the unpaired student’s t-test if variables were normally distributed or otherwise with the Mann–Whitney U test. Dichotomous variables are expressed as absolute values and percentage. Analysis was performed with contingency tables and the Fisher´s exact test. Arrhythmia free survival was analyzed by Kaplan–Meier analysis. The p-value was determined using the Gehan-Breslow-Wilcoxon test and the hazard ratio (HR) was determined with the Mantel–Haenszel method. P-values < 0.05 were considered as statistically significant.

## Results

### Patient characteristics

Between December 2019 and March 2023 a total of 340 patients underwent redo procedures for recurrence of AF using either a LPLD or vHPSD energy settings. The LPLD group consisted of 292 patients (58.2% male; 68.0 ± 11.0 y) and the vHPSD group of 48 patients (62.5% male; 68.0 ± 10.6 y). Baseline characteristics and comorbidities were comparable between groups with exception of COPD (LPLD: 4.8%, vHPSD: 12.5%; *p* = 0.047) and coronary artery disease (LPLD: 19.2%, vHPSD: 37.5%; *p* = 0.008) which were more prevalent in the vHPSD group. In both groups persAF was more prevalent than PAF (LPLD: 66.4%; vHPSD: 70.8%; *p* = 0.6). In each group the average number of prior AF procedures was 1.3 (LPLD: 1.3 ± 0.6; vHPSD: 1.3 ± 0.7; *p* = 0.7) and use of beta-blockers (LPLD: 81.2%; vHPSD: 89.6%; *p* = 0.2) and antiarrhythmic drugs (AADs,LPLD: 31.2%; vHPSD: 31.3%; *p* = 1.0) did not differ significantly between groups. Table 1 displays relevant baseline characteristics and comorbidities.

**Table 1:** Baseline characteristics of the study population

	Total	LPLD	vHPSD	*P*-Value
Sex (male)	200/340 (58.8%)	170/292 (58.2%)	30/48 (62.5%)	0.6
Age	68.0 ± 10.6	68.0 ± 11.0	68.0 ± 10.6	0.8
BMI (kg/m^2^)	26.9 ± 4.4	26.8 ± 4.4	27.1 ± 4.2	0.6
LVEF < 50%	47/324 (14.5%)	36/279 (12.9%)	11/45 (24.4%)	0.1
PersAF	228/340 (67.1%)	194/292 (66.4%)	34/48 (70.8%)	0.6
EHRA				
1	11/340 (3.2%)	9/292 (3.1%)	2/48 (4.2%)	0.7
2a	15/340 (4.4%)	12/292 (4.1%)	3/48 (6.3%)	0.5
2b	146/340 (42.9%)	125/292 (42.8%)	21/48 (43.8%)	> 0.99
3	162/340 (47.6%)	141/292 (48.3%)	21/48 (43.8%)	0.6
4	6/340 (1.8%)	5/292 (1.7%)	1/48 (2.1%)	> 0.99
LA-Diameter (mm)	40.2 ± 6.1	40.2 ± 6.1	40.1 ± 6.7	0.8
Prior AF procedures	1.3 ± 0.6	1.3 ± 0.6	1.3 ± 0.7	0.6
Comorbidities				
Arterial hypertension	235/340 (69.1%)	201/292 (68.8%)	34/48 (70.8%)	0.9
Diabetes	36/340 (10.6%)	30/292 (10.3%)	6/48 (12.5%)	0.6
Hyperlipoproteinemia	106/340 (31.2%)	95/292 (32.5%)	11/48 (22.9%)	0.2
Coronary artery disease	74/340 (21.8%)	56/292 (19.2%)	18/48 (37.5%)	0.008
History of stroke or TIA	33/340 (9.7%)	28/292 (9.6%)	5/48 (10.4%)	0.8
OSAS	43/340 (12.6%)	38/292 (13.0%)	5/48 (10.4%)	0.8
COPD	20/340 (5.9%)	14/292 (4.8%)	6/48 (12.5%)	0.047
PAH	15/340 (4.4%)	12/292 (4.1%)	3/48 (6.3%)	0.5
CIED	38/340 (11.2%)	31/292 (10.6%)	7/48 (14.6%)	0.5
GFR (ml/min; CKD-EPI)	70.3 ± 19.6	70.8 ± 18.5	66.9 ± 25.4	0.3
CHA_2_DS_2_-VASc-Score	2.8 ± 1.7	2.8 ± 1.7	3.1 ± 1.8	0.3
HAS-BLED-Score	0.9 ± 0.7	0.9 ± 0.7	0.9 ± 0.8	0.9
Medication				
Beta-blocker	280/340 (82.4%)	237/292 (81.2%)	43/48 (89.6%)	0.2
AAD	106/340 (31.2%)	91/292 (31.2%)	15/48 (31.3%)	> 0.99
Anticoagulation	308/340 (90.6%)	266/292 (91.1%)	42/48 (87.5%)	0.4

*LPLD* low power long duration, *vHPSD* very high power short duration, *LVEF* left ventricular ejection fraction, *PersAF* persistent atrial fibrillation, *TIA* transient ischemic attack, *OSAS* obstructive sleep apnea syndrome, *COPD* chronic obstructive pulmonary disease, *PAH* pulmonary arterial hypertension, *CIED* Cardiac implantable electronic device, *GFR* glomerular filtration rate, *AAD* antiarrhythmic drug

### Ablation strategies

At the beginning of the procedure, atrial fibrillation was present in 22.3% of patients in the LPLD group and 25.0% in the vHPSD group (*p* = 0.7). More than two thirds of patients showed reconnection of PVs in both groups (LPLD: 67.8%; vHPSD: 70.8%; *p* = 0.7; Table 2). Among patients with PV reconnection comparable proportions of both groups had persAF (LPLD: 61.6%; vHPSD: 61.8%; *p* > 0.9) and LA scarring detected by 3D HD mapping (LPLD: 43.4%; vHPSD: 44.1%; *p* > 0.9). Among patients with durable PVI, persAF was common in both groups (LPLD: 77.7%; vHPSD: 92.8%; *p* = 0.3), along with a high prevalence of left atrial scarring (LPLD: 62.7%; vHPSD: 71.4%; *p* = 0.8).

**Table 2:** Mapping results

	Total	LPLD	vHPSD	*P*-Value
PV reconnection	232/340 (68.2%)	198/292 (67.8%)	34/48 (70.8%)	0.7
LA scar (total)	170/340 (50.0%)	145/292 (49.7%)	25/48 (52.1%)	0.9
- LA scar in patients with PV reconnection	101/232 (43.5%)	86/198 (43.4%)	15/34 (44.1%)	> 0.99
- LA scar in patients with durable PVI	69/108 (63.9%)	59/94 (62.7%)	10/14 (71.4%)	0.8

*LPLD* low power long duration, *vHPSD* very high power short duration, *PVI* pulmonary vein isolation

Ablation strategies for AF consisted of re-isolation of PVs, substrate modification (CFAE/substrate encirclement) and the creation of ablation lines (Table 3). Even though all patients with reconnected PVs received a re-PVI, this was for the minority the only ablation strategy (LPLD: 30.8%; vHPSD: 17.6%; *p* = 0.2). In most cases further ablation strategies were applied. Substrate modification (CFAE ablation/scar encirclement/defragmentation) was performed more often in the vHPSD group (LPLD: 27.7%; vHPSD: 45.8%; *p* = 0.017). Ablation lines were created in more than half of the procedures in both groups (LPLD: 59.9%; vHPSD: 75.0%; *p* = 0.1) and in the vHPSD group more ablation lines were performed per procedure (LPLD: 0.98 ± 0.96; vHPSD: 1.50 ± 1.11; *p* = 0.002). The most prevalent linear lesion was a LA roof line (LPLD: 50.7%; vHPSD: 58.3%; *p* = 0.4), followed by an anterior line, which was more often performed in the vHPSD group (LPLD: 24.7%; vHPSD: 43.8%; *p* = 0.008). An inferior line (LPLD: 21.6%; vHPSD: 37.5%; *p* = 0.027) and Mitral isthmus line were also more often performed in the vHPSD group. If necessary, AT-ablation was added without any difference between groups (LPLD: 17.8%; vHPSD: 22.9%; *p* = 0.4). In the case of typical atrial flutter, additional CTI-ablation was performed with LPLD settings in both groups (LPLD: 17.8%; vHPSD: 27.1%; *p* = 0.2).

**Table 3:** Ablation strategies

	Total	LPLD	vHPSD	*P*-Value
Re-PVI	232/340 (68.2%)	198/292 (67.8%)	34/48 (70.8%)	0.7
Re-PVI only	67/232 (28.9%)	61/198 (30.8%)	6/34 (17.6%)	0.2
Substrate modification	103/340 (30.3%)	81/292 (27.7%)	22/48 (45.8%)	0.017
Procedures with ablation lines	211/340 (62.1%)	175/292 (59.9%)	36/48 (75.0%)	0.1
Number of ablation lines per procedure	1.06 ± 1.00	0.98 ± 0.96	1.50 ± 1.11	0.002
Ablation lines				
Mitral Isthmus	9/340 (2.6%)	4/292 (1.4%)	5/48 (10.4%)	0.004
LA Roof	176/340 (51.8%)	148/292 (50.7%)	28/48 (58.3%)	0.4
Inferior Line	81/340 (23.8%)	63/292 (21.6%)	18/48 (37.5%)	0.027
Anterior line	93/340 (27.4%)	72/292 (24.7%)	21/48 (43.8%)	0.008
AT-ablation	63/340 (18.5%)	52/292 (17.8%)	11/48 (22.9%)	0.4
CTI-ablation*	67/340 (19.7%)	54/292 (18.5%)	13/48 (27.1%)	0.2

*LPLD* low power long duration, *vHPSD* very high power short duration, *Re-PVI* repeat pulmonary vein isolation, *AT* atrial tachycardia, *CTI* cavotricuspid isthmus, *CTI-ablation was performed with LPLD settings in both groups

### Procedural data

**Fig. 1:**
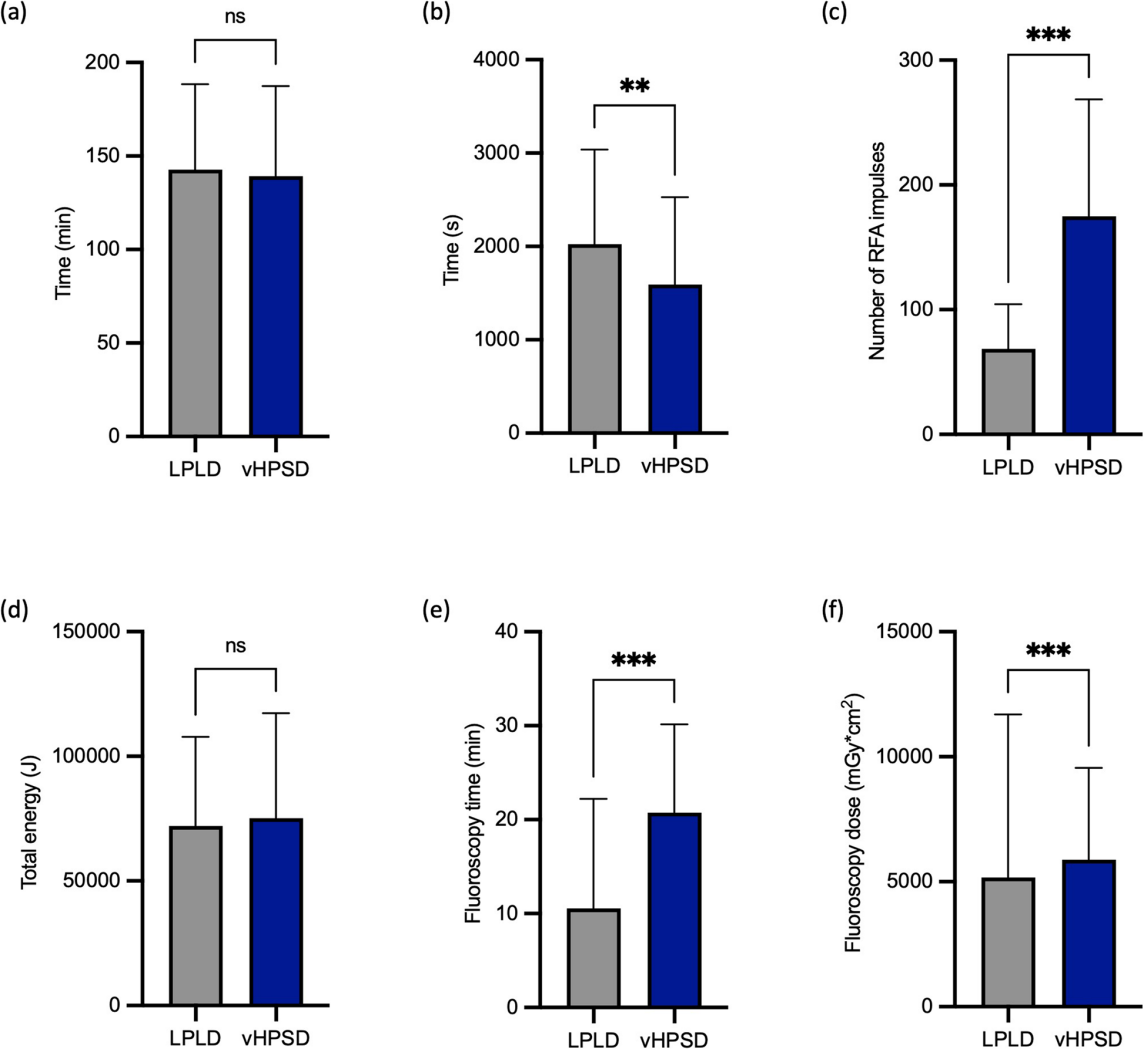
Comparison of procedural parameters between LPLD and vHPSD ablation. **a** Procedure time (skin-to-skin). **b** Ablation time. **c** Number of RFA impulses. **d** Total delivered energy. **e** Fluoroscopy time. **f** Fluoroscopy dose. Bars represent mean ± SD

The total procedure time (skin-to-skin) was comparable between groups (*p* = 0.7; Fig. 1a, Table 4). The RF delivery time was significantly shorter in the vHPSD group than in the LPLD group (LPLD: 2023.5 ± 1014.0 s; vHPSD: 1591.4 ± 935.7 s; *p* = 0.006; Fig. 1b), even though in the vHPSD group per procedure more ablation lines were performed, more patients received substrate modification and more RFA impulses were applied (LPLD: 68.7 ± 35.8; vHPSD: 174.8 ± 93.8; *p* < 0.001; Fig. 1c). The total energy was comparable and fluoroscopy dose and time were longer than in the LPLD group (Figs. 1d-f, Table 4).

**Table 4:** Procedural characteristics

	**Total**	**LPLD**	**vHPSD**	***P*-Value**
Procedure time (min, skin-to-skin)	142.3 ± 46.1	142.8 ± 45.8	139.2 ± 48.2	0.7
RFA ablation time (s)	1962.1 ± 1013.3	2023.5 ± 1014.0	1591.4 ± 935.7	0.006
RFA impulses	83.8 ± 60.8	68.7 ± 35.8	174.8 ± 93.8	< 0.001
RFA total energy (J)	72,559.7 ± 36,631.4	72,118.3 ± 35,710.3	75,226.5 ± 42,091.4	0.6
Fluoroscopy time (min)	12.0 ± 11.9	10.5 ± 11.7	20.8 ± 9.4	< 0.001
Fluoroscopy dose (mGy $\bullet$ cm^2^)	5273.7 ± 6192.5	5172.4 ± 6519.6	5877.3 ± 3676.3	< 0.001

*LPLD* low power long duration, *vHPSD* very high power short duration*, RFA* radiofrequency ablation

### Outcome

**Fig. 2:**
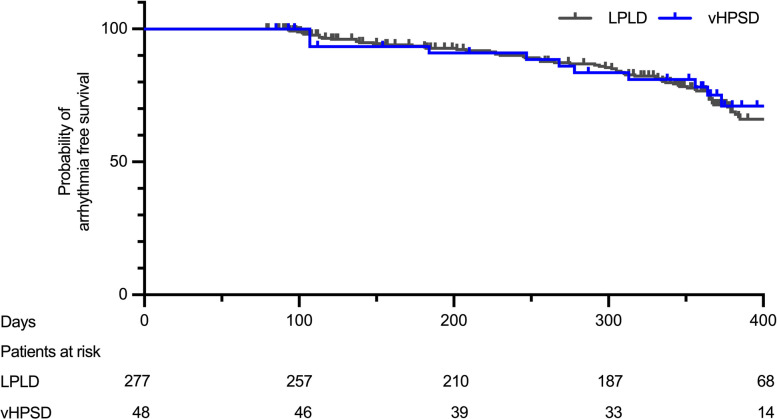
Arrhythmia-free survival after redo ablation. Kaplan–Meier curves of arrhythmia-free survival comparing LPLD and vHPSD ablation strategies

After a blanking period of eight weeks [1] for 277 patients (94.9%) of the LPLD group and 48 patients (100%) of the vHPSD group a follow-up was available. Median follow-up was after 361 days. In the survival analysis the arrhythmia free survival did not differ between groups (*p* = 0.8; HR 0.87; 95% CI 0.52—1.47) and was overall 65.7% in the LPLD vs. 68.8% in the vHPSD group (Fig. 2).

### Safety

As major complication one pericardial tamponade requiring pericardiocentesis occurred in the LPLD group. Moreover, one patient presented with a subacute pericarditis in the LPLD group requiring pericardiocentesis of a serious pericardial effusion and anti-inflammatory therapy. Two TIAs but no stroke were observed in the LPLD group and no thromboembolic event in the vHPSD group. Three patients developed a sinus arrest in the LPLD, and one patient in the vHPSD group. A phrenic nerve palsy, atrioesophageal fistula or pulmonary vein stenosis were not reported. Complications of the access site (hematoma, pseudoaneurysm, AV fistula) were comparable between groups. Overall, there was no statistically significant difference between complications in LPLD or vHPSD ablation settings (*p* = 0.3) indicating comparable complications (Table 5).

**Table 5:** Safety

	Total	LPLD	vHPSD	*P*-Value
Hematoma	6/340 (1.8%)	4/292 (1.4%)	2/48 (4.2%)	0.2
Pseudoaneurysm	1/340 (0.3%)	1/292 (0.3%)	0/48 (0%)	> 0.99
AV fistula	1/340 (0.3%)	1/292 (0.3%)	0/48 (0%)	> 0.99
Pericardial effusion	3/340 (0.9%)	2/292 (0.7%)	1/48 (2.1%)	0.4
Pericardial tamponade	1/340 (0.3%)	1/292 (0.3%)	0/48 (0%)	> 0.99
Pericarditis	1/340 (0.3%)	1/292 (0.3%)	0/48 (0%)	> 0.99
Stroke	0/340 (0%)	0/292 (0%)	0/48 (0%)	-
TIA	2/340 (0.6%)	2/292 (0.7%)	0/48 (0%)	> 0.99
Phrenic nerve palsy	0/340 (0%)	0/292 (0%)	0/48 (0%)	-
Sinus arrest	4/340 (1.2%)	3/292 (1.0%)	1/48 (2.1%)	0.5
Atrioesophageal fistula	0/340 (0%)	0/292 (0%)	0/48 (0%)	-
PV stenosis	0/340 (0%)	0/292 (0%)	0/48 (0%)	-
Total	19/340 (5.6%)	15/292 (5.1%)	4/48 (8.3%)	0.3

*LPLD* low power long duration, *vHPSD* very high power short duration, *RFA* radiofrequency ablation, *TIA* transient ischemic attack, *PV* pulmonary vein

## Discussion

### Main findings

There is growing evidence that vHPSD is safe and has the potential to significantly shorten the ablation time for PVI compared with LPLD settings [7, 8, 14, 15]. However, data on lesion sets beyond PVI remain limited. In our study of re-ablation procedures, the RF application time was shorter in the vHPSD group, despite more ablation lines per procedure and a higher proportion of patients requiring substrate modification. This suggests that vHPSD may improve time efficiency even in complex cases. Fluoroscopy dose and duration were higher in the vHPSD group. A possible explanation is increased catheter manipulation required for more extensive ablation and additional linear lesions. Furthermore, when using the FlexAbility catheter without contact-force sensing as required for vHPSD application, operators may have relied more on fluoroscopic verification of catheter position despite the use of 3D mapping. These factors may also explain the lack of a significant reduction in overall procedure time. With increased operator experience and exclusive use of contact force measuring catheters, it is likely that fluoroscopy time and overall procedure time can also be reduced, further maximizing the advantages of vHPSD. Although overall procedure duration was similar between groups, vHPSD provided a clear procedural benefit by markedly reducing RF delivery time, even in cases requiring extensive linear lesions and substrate modification. The approach proved feasible in complex redo procedures comparable to LPLD, despite the use of catheters without contact force sensing. These findings support vHPSD as an efficient alternative energy setting in redo ablation rather than a superior strategy in terms of clinical outcome.

There are only a few trials investigating HPSD settings for anatomical ablation lines, and most have used 50 W rather than a vHPSD setting (≥ 70 W). Zanchi et al. demonstrated the safety of anterior and roof line ablation with AI-guided ablation at 50 W, achieving a high first-pass conduction block rate of 75% for the anterior line and 82% for the roof line [16]. Winkle et al. showed that posterior wall isolation (PWI) and roof line ablation at 50 W were safe; however, PWI was associated with worse outcomes in patients with persAF and longstanding persistent atrial fibrillation [17]. Conti et al. studied PVI with posterior wall isolation guided by LSI at 50 W, showing that HPSD settings reduced procedure and ablation times without differences in safety or outcomes [18]. Similarly, Yavin et al. successfully performed PVI with additional CTI ablation using HPSD settings (50 W for 15 s) [19]. In our study, we avoided vHPSD settings for CTI ablation due to the unique tissue characteristics and the shallower lesions expected with vHPSD. However, our results suggest that left atrial ablation lines with even higher energy settings in a vHPSD setting (≥ 70 W) are safe and feasible.

In our study cohort, the majority of patients suffered from persAF and a relevant number of patients had a scarred LA, which are both predictors of an unfavorable outcome [20]. Nevertheless overall freedom of any arrhythmia was 65.7% vs. 68.8% after a median follow up of 361 days which was comparable between groups. Of note, only 11.2% of patients had continuous rhythm monitoring through a cardiac device, so short and asymptomatic episodes of arrhythmia recurrence may have remained undetected. However, the observed outcomes remain consistent and comparable between groups. A study from Ptaszek et. al. demonstrated no difference in pulmonary vein isolation rate between HPSD (50 W for 10 s) and LPLD in a repeat procedure 28 days after the PVI, indicating comparable durability of lesions [21]. Supporting this finding, a MRI study proved transmurality and durability of PV scars following HPSD PVI [22]. In the FAST and FURIOUS redo study, even a higher durability of HPSD PVI compared to LPLD settings was shown [23] and Kottmaier et. al. demonstrated higher arrhythmia free survival of a vHPSD group (70 W) compared to a LPLD group (HPSD: 83.1%; LPLD: 65.1%) [8]. That study included only PAF patients and analyzed first ablation procedures which could explain why the recurrence of atrial arrhythmia was higher in our study. Importantly, the best strategy beyond PVI remains unclear, which is why the ablation approaches in our study were heterogeneous. In the PARTY-PVI study, ablation lines, electrogram-guided ablation, and PV-dependent ablation showed similar results [24]. Moreover, a recent study found that CS-based ablation offered no superiority [25].

Main concerns for vHPSD are steam pops leading to thrombi and thromboembolic events and pericardial tamponades by cardiac perforation. The slightly, but not statistically significantly, higher complication rate in the vHPSD cohort was driven primarily by minor vascular access-site events, which are unlikely to be related to the ablation modality, as identical access techniques and sheaths were used in both groups. Importantly, serious complications were rare and occurred at comparable rates. The POWER FAST III trial compared a vHPSD group (70 W) with a LPLD group (25–40 W) which are similar power settings to our study groups. The study found a high incidence of postprocedural cerebral lesions for vHPSD in MRI [26]. However, none of those were clinically apparent. The used catheter design (irrigation type) in the POWER FAST III trial might not have been ideal for the chosen energy setting. In our study cohort no stroke was observed. Two TIAs occurred in the LPLD but no thromboembolic events in the vHPSD group. Most other studies did comparably not find an increased incidence of clinical apparent thromboembolic events [8, 14]. However, we did not perform a systematic screening for subclinical thromboembolic lesions. Given the limited cohort size, particularly in the vHPSD group, larger studies are needed to more definitively assess potential differences in safety.

Esophageal injury and atrioesophageal fistula are a severe but rare [27] potential complication of RFA due to thermal injury. A study which investigated 574 patients who underwent HPSD (50 W) and 113 who underwent LPLD (≤ 35 W) with MRI imaging within 24 h post-ablation found no difference in the esophageal LGE patterns and no occurrence of atrioesophageal fistulas [28]. In the POWER-AF study an ulcerative esophageal perforation requiring stenting was described after ablation with a setting of 45 W and a CLOSE guided protocol following ablation at the posterior wall [29]. In our cohort no esophageal injury occurred but due to the rare occurence a larger study cohort would be necessary to exclude an association to vHPSD.

For the first PVI vHPSD might not be the fastest option since 3D mapping is required which takes additional time as opposed to single-shot devices. In comparison with PFA-PVI and Cryoballoon PVI procedure time is usually longer with HPSD [9, 30]. However, even though single-shot devices can be used in selected cases and 3D mapping workflows have been proposed [31], their design generally limits the creation of extended linear lesions or precise substrate modification which restricts their use as alternative to RFA in redo-procedures. Single tip PFA devices and those with dual energy technology have recently become available and will evolve as an alternative to RF ablation for these ablation concepts [32, 33].

Recent data indicate that vHPSD lesion formation may vary depending on tissue thickness, with particularly challenging areas along the anterior left atrial wall where lesions can be comparatively shallow. Although some studies have shown excellent acute isolation rates with vHPSD approaches [23], others have reported lower efficacy in these regions [34, 35]. This variability in lesion depth should be taken into account when interpreting our findings.

### Study limitations

As a retrospective cohort study this study has several limitations. One important limitation is that there was no defined ablation protocol and ablation strategies were diverse. Moreover, choice of ablation strategy was at operator’s discretion and different ablation catheters were used. Since the atrial substrate is highly individual, ablation targets and the required extent of ablation were heterogenous, which makes it difficult to compare ablation parameters. Because the vHPSD group underwent more extensive substrate modification, including a higher number of linear lesions, this disparity may have impacted arrhythmia recurrence and should be considered a relevant confounding factor. We acknowledge that procedures were performed without ICE and under conscious sedation, which represents the routine workflow at our center. While this approach is well established and was safe in our cohort as well as in clinical practice, the absence of additional imaging and controlled ventilation may be regarded as a limitation in the context of vHPSD. Furthermore, our study is a small single center study with a limited number of patients and the unequal group sizes represent an additional limitation that may influence the interpretation of the results. There is no doubt that further studies, preferably RCTs, are required to validate the results.

## Conclusion

This study suggests that a vHPSD (≥ 70 W) approach for repeat ablation of AF including substrate modification and linear ablation strategies is feasible. Complications and outcome appear to be comparable between LPLD and vHPSD settings. However, to validate these results further research, preferentially randomized controlled trials (RCTs) are required.

## Data Availability

The datasets generated and analyzed during the current study are not publicly available due to data protection and privacy regulations but are available from the corresponding author on reasonable request.

## Abbreviations

AAD: Antiarrhythmic drug
ACT: Activated clotting time
AF: Atrial fibrillation
AI: Ablation index
AT: Atrial tachycardia
AV: Arteriovenous
BMI: Body mass index
CA: Catheter ablation
CFAE: Complex fractionated atrial electrogram
CKD-EPI: Chronic Kidney Disease Epidemiology Collaboration
CI: Confidence interval
CIED: Cardiac implantable electronic device
COPD: Chronic obstructive pulmonary disease
CS: Coronary sinus
CTI: Cavotricuspid isthmus
DOAC: Direct oral anticoagulant
ECG: Electrocardiogram
EHRA: European Heart Rhythm Association
GFR: Glomerular filtration rate
HD: High-density
HR: Hazard ratio
HPSD: High power short duration
INR: International normalized ratio
LA: Left atrium / left atrial
LGE: Late gadolinium enhancement
LPLD: Low power long duration
LSI: Lesion index
LVEF: Left ventricular ejection fraction
MRI: Magnetic resonance imaging
OSAS: Obstructive sleep apnea syndrome
PAF: Paroxysmal atrial fibrillation
PAH: Pulmonary arterial hypertension
PersAF: Persistent atrial fibrillation
PFA: Pulsed field ablation
PV: Pulmonary vein
PVI: Pulmonary vein isolation
PWI: Posterior wall isolation
RCT: Randomized controlled trial
RFA: Radiofrequency ablation
SR: Sinus rhythm
TEE: Transesophageal echocardiography
TIA: Transient ischemic attack
TSP: Transseptal puncture
TTE: Transthoracic echocardiography
UFH: Unfractionated heparin
vHPSD: Very high power short duration
